# Clinical outcomes of open versus laparoscopic inguinal hernia mesh repair in Palestine: a multicenter retrospective cohort study

**DOI:** 10.1186/s12893-026-04029-1

**Published:** 2026-07-10

**Authors:** Iyad M. Ballas, Husam N. Abualwafa, Ibrahim H. AL-Shaer, Ibrahim Aljamal, Ahmad Tabhoub, Saja Nazzal, Mahdi Aljamal

**Affiliations:** 1 Faculty of Medicine, Arab American University, Jenin, Palestine; 2 Department of General Surgery, AL-Makassed Hospital, Jerusalem, Palestine; 3 Department of Medicine, Faculty of Medicine and Health Sciences, An-Najah National University, Nablus, Palestine; 4 Department of General Surgery, Faculty of Medicine, Arab American University, Jenin, Palestine

**Keywords:** Inguinal hernia, Hernia repair, Laparoscopic surgery, Open surgery, Mesh repair, Retrospective cohort, Palestine

## Abstract

**Background:**

Inguinal hernia repair is one of the most frequently performed surgical procedures worldwide, accounting for approximately 75% of abdominal wall hernias, and predominantly occurring in males. Although it is a common procedure with generally favourable outcomes, the postoperative outcomes, such as chronic pain, recurrence, and surgical complications, remain important concerns. The aim of this study was to compare short-term postoperative outcomes and complications between open and laparoscopic inguinal hernia mesh repair.

**Method:**

A retrospective cohort study was conducted to compare the outcomes between open vs. laparoscopic inguinal hernia mesh repair. All patients who underwent inguinal hernia mesh repair in 3 hospitals from 2017 to 2025 were collected. Patients were divided into groups based on surgical technique (open repair vs. laparoscopic repair). Demographic data, operative time, length of hospital stay, postoperative outcomes including surgical complications (hematoma, seroma, surgical site infection, hydrocele, recurrence), and general postoperative complications (urinary retention and postoperative nausea/vomiting) were extracted and analysed. A P-value < 0.05 is considered statistically significant.

**Results:**

A total of 609 patients underwent inguinal hernia repair with mesh. Open repair was more prevalent (77.2%). Reducible and uncomplicated hernias were significantly associated with laparoscopic repair (*p* < 0.001, *p* = 0.006), while irreducible and strangulated hernias were predominantly managed with open surgery. Patients undergoing laparoscopic repair were younger (median 51.5 vs. 55 years, *p* = 0.001) and had shorter operative duration (median 101.5 vs. 113 min, *p* = 0.001). Urinary retention occurred more frequently after open repair (5.5% vs. 0.7%, *p* = 0.015), whereas postoperative nausea and vomiting were more common following laparoscopy (2.2% vs. 0.2%, *p* = 0.039).

Overall postoperative complications were low and comparable between the two approaches (12.6% vs. 14.4%, *p* = 0.572), and early documented recurrence during short-term follow-up was uncommon and did not differ significantly between groups; however, the follow-up duration was insufficient to reliably assess true recurrence rates. (1.1% vs. 1.4%, *p* = 0.709).

**Conclusion:**

Both open and laparoscopic inguinal hernia repair demonstrated favourable safety profiles in this cohort and were associated with low rates of short-term postoperative complications. However, the short follow-up duration limits assessment of long-term outcomes such as recurrence, chronic postoperative pain, and mesh-related complications.

## Introduction

### Background

An inguinal hernia is the protrusion of abdominal content through a defect in the abdominal wall into the inguinal canal [1]. An indirect hernia occurs when abdominal contents protrude through the internal ring [2] and follow the inguinal canal, and a direct hernia occurs when the abdominal content pushes directly through a weakened area of the posterior wall of the inguinal canal [3]. Globally, 20 million inguinal hernia operations are performed annually, making it the most frequently repaired by surgical approach [4, 5]. It is approximately 75% from all abdominal wall hernias and occurs more frequently in males (27%) than in females (3%) [6].

The large number of hernia repair procedures significantly contributes to healthcare resource utilization and economic costs, and exceeds 3 billion euros annually in both low- and high-income settings [7, 8]. Although inguinal hernia repair is regarded as a routine surgical procedure, there is ongoing debate about the most effective surgical approach (open, laparoscopic, robotic), taking into account outcomes, cost, and patient-specific factors [3].

Although several randomized trials and meta-analyses have compared open and laparoscopic inguinal hernia repair, most evidence originates from Europe, North America, and East Asia. Data from the Middle East remain limited, particularly from Palestine. Differences in healthcare infrastructure, surgical expertise, patient characteristics, and referral patterns may influence surgical outcomes and limit the direct applicability of international evidence to local practice. Therefore, this study aimed to provide multicenter real-world data from Palestinian hospitals and evaluate short-term postoperative outcomes following open and laparoscopic mesh repair.

### Literature review

Inguinal hernia repair is one of the most commonly performed surgical procedures worldwide. Over the past decade, open mesh repair (classically the Lichtenstein technique) and laparoscopic mesh repair (TAPP and TEP) have been the dominant operative strategies. Recent evidence and guideline updates from the European Hernia Society and the HerniaSurge Group provide current comparative insights into outcomes and technique selection [1].

The Lichtenstein tension-free mesh repair remains the most widely used open technique. Although originally described several decades ago, it continues to be supported as a standard approach in more recent guideline updates due to its simplicity, reproducibility, and effectiveness for primary unilateral hernias. It remains especially relevant in settings with limited laparoscopic resources or in patients unsuitable for laparoscopy [1, 9].

Minimally invasive approaches have increasingly been used over the last decade. The two main laparoscopic techniques are Transabdominal Preperitoneal Repair (TAPP) and Totally Extraperitoneal Repair (TEP). Recent comparative studies and meta-analyses demonstrate that laparoscopic approaches are associated with several perioperative benefits compared with open repair [9].

Based on the studies, there were different advantages of the laparoscopic approach as patients have Less postoperative pain. Multiple randomized and observational studies report significantly reduced early postoperative pain scores in patients undergoing TAPP/TEP versus open repair. Furthermore, it was associated with Faster functional recovery. Patients tend to return to daily activities and work sooner. Its advantage is also apparent in repairing bilateral hernia. Both sides can be accessed with the same laparoscopic ports, avoiding bilateral incisions [1, 9].

Despite these advantages of the laparoscopic approach, open techniques still have multiple advantages and remain a simpler technique. Open repair is technically less complex and does not require specialized laparoscopic skills. And based on the cost, it has a lower cost. Open repair generally incurs lower direct costs due to the absence of laparoscopic equipment. And it is more suitable for complicated cases, e.g., large scrotal or strangulated hernias may be safer via open repair [1, 10].

Despite numerous studies comparing open and laparoscopic inguinal hernia repair, controversy remains regarding operative time, postoperative complications, and recurrence rates. While some studies report advantages of laparoscopic repair in terms of postoperative pain and recovery, findings regarding complication and recurrence rates remain inconsistent [11, 12].

Furthermore, major evidence arises from studies performed in Europe, the United States, and East Asia with restricted data from the Middle East and limited outcomes in specific region as in Palestine populations. This geographic discrimination emphasizes a distinct gap in knowledge, which restricts the generalizability of findings to local surgical practice and patient populations.

### Problem statement

Most available evidence comparing open and laparoscopic inguinal hernia repair originates from Europe, North America, and East Asia. Data from Palestine and the wider Middle East remain limited. Differences in patient characteristics, healthcare infrastructure, surgeon expertise, and hospital resources may influence surgical outcomes and limit the generalizability of international findings. Therefore, this multicenter study aimed to compare short-term postoperative outcomes following open and laparoscopic mesh repair of inguinal hernia in Palestinian hospitals.

### Study objectives

#### Primary objective

To compare short-term postoperative outcomes and complications between open and laparoscopic mesh repair of inguinal hernia in Palestinian hospitals.

#### Secondary objectives

- To find descriptive data in patients with inguinal hernia in Palestine.
- To assess the association between the type of surgery and postoperative complications.
- To assess the association between the type of surgery and intraoperative complications.
- To assess the association between the type of surgery and socio-demographic data.

## Methods

### Study design

This study was designed as a retrospective cohort study evaluating outcomes of open and laparoscopic inguinal hernia repair in three hospitals in the West Bank between January 2017 and December 2025. This study was conducted and reported in accordance with the Strengthening the Reporting of Observational Studies in Epidemiology (STROBE) guidelines.

### Study setting

This study included three hospitals in the West Bank ( Ibn Sina Specialized Hospital, Jenin Governmental Hospital, and Makassed Islamic Charitable Hospital), two of which are private hospitals and one governmental hospital. The hospitals are located in the northern and central regions of the West Bank, ensuring representation from different healthcare sectors and geographic areas.

### Study population

We included patients who met the inclusion criteria and were operated on with inguinal hernia repair with mesh, either open or laparoscopic surgery, from 2017 to 2025. Both male and female adult patients were eligible for inclusion if they fulfilled the study criteria.

#### Inclusion criteria

- Adult patients (> 18 years)
- Diagnosed with a primary unilateral or bilateral inguinal hernia
- Underwent open or laparoscopic mesh repair
- Complete medical records, including operative notes and follow-up data, were required

#### Exclusion criteria

- Patient under 18 years old
- Non-mesh repairs of inguinal hernias
- Recurrent inguinal hernia without documented data
- Femoral hernia (excluded because femoral hernias differ from inguinal hernias in anatomy, epidemiology, operative management, and postoperative outcomes, which could introduce clinical heterogeneity)
- Missing operative data
- Any laparoscopic hernia repair converted to open repair

### Sample size

This was a retrospective comparative cohort study. All adult patients who underwent inguinal hernia repair with mesh (laparoscopic or open) at [Ibn Sina specialized hospital, Jenin governmental hospital, and Makassed Islamic charitable hospital] between [1/1/2017] and [25/12/2025] were included. No a priori sample size calculation was performed because the study used total population sampling of all eligible cases during the study period.

### Surgical technique

Open repair was predominantly performed using the Lichtenstein tension-free mesh technique through an anterior groin approach. After reduction of the hernia sac, a mesh was placed over the posterior wall of the inguinal canal and secured according to the surgeon’s preference. Laparoscopic repair included both transabdominal preperitoneal (TAPP) and totally extraperitoneal (TEP) approaches. In both techniques, mesh was placed in the preperitoneal space to reinforce the myopectineal orifice. Allocation to open or laparoscopic repair was not randomized. The choice of surgical approach was determined by the treating surgeon based on patient characteristics, hernia complexity, surgeon expertise, availability of laparoscopic facilities, and institutional practice. Because of the retrospective multicenter design, complete standardization of mesh fixation techniques and surgeon-specific technical preferences could not be verified.

### Data collection

A data collection sheet was created to include all variables related to our research (socio-demographic, clinical, preoperative, and outcomes). These data were extracted from medical records, operative reports, and clinic follow-up notes, and were kept saved without name or hospital id in a password-protected file, only accessed by the authors. Pain scores were extracted from the medical records and were recorded using a numerical rating scale (NRS) ranging from 0 (no pain) to 10 (worst imaginable pain).

### Statistical analysis

Statistical analysis was carried out using SPSS version 27. Descriptive statistics were employed to describe sociodemographic factors and perioperative data. Categorical variables were described using frequency and percentage. Continuous variables were described using median and interquartile range (IQR) since the data were not normally distributed, as evidenced by the Shapiro-Wilk test.

The Chi-square test was employed to evaluate associations between categorical variables and the type of surgery. The Fisher’s exact test was used when the expected cell count was less than 5. The Mann-Whitney U test was employed to compare groups for continuous variables that were not normally distributed. A p-value of less than 0.05 was considered statistically significant for all tests.

Denominators differed for variables due to missing data.

### Ethical approval

The study was conducted after ethical approval from the IRB at Arab American University, before data collection. Patient data were collected from their medical records. Each patient was given a special code, and stored without patient identifiers in password protected file, only accessed by the authors.

## Results

### Descriptive analysis

Categorical variables were presented with frequencies and percentages (Table 1), and continuous variables were summarized by median and interquartile ranges (IQRs) due to non-normal distribution (Table 2).

**Table 1:** Frequencies of categorical variables

Variable	Category	Frequency	Percentages %
Gender (*n* = 609)	Male	581	95.4
	Female	28	4.6
	Total	609	100
Smoking status (*n* = 593)	Smoker	447	73.4
	Never	98	16.1
	Ex-smoker	48	8.1
	Total	593	97.4
Social status (*n* = 609)	Single	51	8.4
	Married	558	91.6
	Total	609	100
Co-morbidities (*n* = 609)	No	352	57.8
	Yes	257	42.2
	Total	609	100
Hypertension (*n* = 609)	No	463	76.0
	Yes	146	24.0
	Total	609	100
Diabetes (*n* = 609)	No	517	84.9
	yes	92	15.1
	Total	609	100
Ischemic heart disease (*n* = 609)	No	562	92.3
	Yes	47	7.7
	Total	609	100
Heavy lifting (*n* = 609)	No	533	87.5
	Yes	76	12.5
	Total	609	100
Past surgical history (*n* = 609)	No	283	46.5
	Yes	326	53.5
	Total	609	100
Past groin surgery (*n* = 609)	No	480	78.8
	Yes	129	21.2
	Total	609	100
Use of anticoagulants/anti-platelet (*n* = 609)	No	537	88.2
	Yes	72	11.8
	Total	609	100
Type of hernia (*n* = 608)	Direct	257	42.2
	Indirect	305	50.1
	Pantaloon	46	7.6
	Total	608	99.8
Unilateral/ bilateral (*n* = 609)	Unilateral	452	74.2
	Bilateral	157	25.8
	Total	609	100
Right or left (*n* = 609)	Right	334	54.8
	Left	275	45.2
	Total	609	100
Reducible/ irreducible (*n* = 609)	Reducible	554	91.0
	Irreducible	55	9.0
	Total	609	100
Presence of complications (*n* = 609)	No	563	92.4
	Yes	46	7.6
	Total	609	100
Incarceration (*n* = 609)	No	583	95.7
	Yes	26	4.3
	Total	609	100
Obstruction (*n* = 609)	No	601	98.7
	Yes	8	1.3
	Total	609	100
Strangulation (*n* = 609)	No	596	97.9
	Yes	13	2.1
	Total	609	100
Name of hospital (*n* = 609)	Governmental	152	25.0
	Private	457	75.0
	Total	609	100
Type of procedure (*n* = 609)	Open	470	77.2
	Laparoscopic	139	22.8
	Total	609	100
Laparoscpic Approach	TAPP	112	18.4
	TEP	27	4.4
	NOT APPLICABLE	469	77.0
	Total	609	99.8
Mesh used (*n* = 609)	yes	609	100
Mesh type (*n* = 608)	Polypropylene mesh	532	87.3
	3D mesh	76	12.5
	Total	608	99.8
Use of prophylactic antibiotic (*n* = 609)	No	19	3.1
	Yes	590	96.9
	Total	609	100
Intra operative complications	No	609	100
Nausea and vomiting (*n* = 609)	No	605	99.3
	Yes	4	0.7
	Total	609	100
Urinary retention (*n* = 609)	No	582	95.6
	Yes	27	4.4
	Total	609	100
Outcomes / complications (*n* = 609)	No	530	87.0
	One complication	72	11.8
	Two complications	6	1.0
	Three complications	1	0.2
	Total	609	100
Surgical site infection (*n* = 609)	No	604	99.2
	Yes	5	0.8
	total	609	100
Hematoma (*n* = 609)	No	592	97.2
	Yes	17	2.8
	Total	609	100
Seroma (*n* = 609)	No	599	98.4
	Yes	10	1.6
	Total	609	100
Mesh infection (*n* = 609)	No	609	100
	Yes	0	0.0
	Total	609	100
Scrotal edema (*n* = 609)	No	600	98.5
	Yes	9	1.5
	Total	609	100
Testicular complications (*n* = 609)	No	608	99.8
	Yes	1	0.4
	total	609	100
Recurrence of hernia (*n* = 608)	No	601	96.7
	Yes	7	1.15
	Total	608	99.8
Post op pain (*n* = 609)	No	589	96.7
	Yes	20	3.3
	Total	609	100
Hydrocele (*n* = 609)	No	601	98.7
	Yes	8	1.3
	Total	609	100
Other complications (*n* = 492)	No	482	79.1
	Yes	10	1.6
	Total	492	80.8

Percentages were calculated using available data for each variable. Denominators vary because of missing data

**Table 2:** Continuous variable descriptive

Variable	Median	Interquartile ranges (IQ)
Age(years)	64.5	51.25-74.0
Symptoms duration of hernia in months	12	2–36
Preoperative pain out of 10	1.0	0–2
Postoperative pain	0	0–2
Length of hospital stay in days	1	1–2
Last follow up in days	7	5-16.5
Duration of surgery in minutes	107.5	62.5-123.75

#### Sociodemographic characteristics

A total of 609 patients were included in the study. The median age was 64.5 years (interquartile range [IQR]: 51.25–74.0), with ages ranging from 18 to 80 years. The majority of participants were male (581/609, 95.4%), while 28 patients (4.6%) were female; of those patients, 91.6% were married. Regarding smoking status, 73.4% of participants were smokers.

Overall, 42.2% of patients had at least one comorbidity. Hypertension was present in 24.0%, diabetes mellitus in 15.1%, and ischemic heart disease in 7.7%.

In terms of occupational and lifestyle factors, 12.5% reported heavy lifting. A history of previous surgical procedures was noted in 53.5% of participants, and 21.2% had undergone prior groin surgery. All participants were diagnosed with an inguinal hernia and underwent surgical repair on either an elective or emergency basis.

#### Clinical presentation prior to surgery

The median duration of symptoms was 12 months (IQR: 2–36 months). The median preoperative pain score was low, with a median pain score of 1.0 out of 10 (IQR: 0–2). At presentation, 11.8% of patients were receiving antiplatelet and/or anticoagulant therapy.

Most hernias were unilateral (74.2%) and more commonly right-sided (54.8%). Indirect hernias accounted for 50.1% of cases. The majority were reducible (91.0%) and uncomplicated (92.4%).

Complications were observed in 7.6% of cases. Incarceration occurred in 4.3%, strangulation in 2.1%, and bowel obstruction in 1.3%.

#### Perioperative variables and descriptive analysis

All 609 patients underwent inguinal hernia repair. The majority of procedures were performed in private hospitals (75.0%). Open repair was the predominant technique (77.2%), while laparoscopic repair accounted for 22.8% of cases. Among laparoscopic repairs, 112 (80.6%) were performed using the TAPP approach and 27 (19.4%) using the TEP approach. Of the 28 female patients treated with hernia repair, 9 of them were operated on with Laparoscopic hernia repair, and 581 male patients were operated on, with 130 of them undergoing a laparoscopic approach.

Mesh repair was utilized in all cases. Polypropylene mesh was used in 87.3% of procedures. Prophylactic antibiotics were administered in 96.9% of patients.

No intraoperative complications were reported. Urinary retention was the most common postoperative complication (4.4%). Surgical site infection occurred in 0.8%, hematoma in 2.8%, seroma in 1.6%, scrotal edema in 1.5%, hydrocele in 1.3%, and testicular complications in 0.4%. Hernia recurrence was reported in 3.3% of patients.

The median operative time was 107.5 min (IQR: 62.5–123.75), and the median hospital stay was 1 day (IQR: 1–2 days). The median follow-up duration was 7 days (IQR: 5–16.5 days).

### Comparing outcomes of open and laparoscopic approaches to inguinal hernia repair with mesh

An association analysis was performed to evaluate the relationship between categorical clinical variables and the type of surgical approach (open versus laparoscopic repair) using the Chi-square test, with Fisher’s exact test applied when appropriate. Continuous variables were compared using the Mann–Whitney U test.

The type of inguinal hernia (direct, indirect, and pantaloon) was significantly associated with the surgical approach (*p* = 0.037). Direct hernias were relatively more frequent in the laparoscopic group (50.0%), whereas indirect hernias were more commonly managed with open repair (53.0%).

Hernia reducibility demonstrated a strong statistically significant association with the type of surgery (*p* < 0.001). Irreducible hernias were predominantly managed with open repair (11.3% vs. 1.4%), whereas laparoscopic repair was more frequently performed in reducible cases (98.6%).

The presence of complications was significantly associated with the surgical approach (*p* = 0.006), with complicated hernias more commonly treated using open repair (9.1% vs. 2.2%). Strangulation showed a statistically significant association with the surgical approach based on Fisher’s exact test (*p* = 0.047), as all strangulated cases were treated with open repair and none underwent laparoscopic repair.

Postoperative nausea and vomiting were significantly associated with the type of surgery (*p* = 0.039), occurring more frequently in the laparoscopic group (2.2% vs. 0.2%). Urinary retention was also significantly associated with surgical approach (*p* = 0.015), with most cases occurring after open repair (5.5% vs. 0.7%). Additional analysis demonstrated that among patients undergoing open repair, postoperative urinary retention occurred in 12 of 114 patients (10.5%) who received spinal anesthesia compared with 8 of 210 patients (3.8%) who received general anesthesia (*p* = 0.027). Postoperative overall complications were not significantly associated with the type of surgery (*p* = 0.572).

Regarding continuous variables, age differed significantly between the two groups (Mann–Whitney U test, *p* = 0.001), with a median age of 55 years (IQR: 41–66) in the open repair group and 51.5 years (IQR: 38–59) in the laparoscopic group. Similarly, the duration of surgery was significantly different between the two approaches (Mann–Whitney U test, *p* = 0.0011), with a median duration of 113 min (IQR: 88–140) for open repair and 101.5 min (IQR: 78–124.5) for laparoscopic repair. All other variables not mentioned demonstrated no statistically significant association with the type of surgery (*p* > 0.05) (Tables 3, 4 and 5).

**Table 3:** Association between categorical and type of surgery (chi-square/Fisher test)

Variable		Open herniorrhaphy *N*(%)	Laparoscopic herniorrhaphy *N*(%)	Total	*P*-value
Type of inguinal hernia	Direct	188 (40.0)	69 (50.0)	257 (42.3)	0.037
	Indirect	249 (53.0)	56 (40.6)	305 (50.2)	
	Pantaloon	33 (7.0)	13 (9.4)	46 (7.6)	
Reducible_Irreducible	Reducible	417 (88.7)	137 (98.6)	554 (91.0)	< 0.001
	Irreducible	53 (11.3)	2 (1.4)	55 (9.0)	
Presence of complication	No	427 (90.9)	136 (97.8)	563 (92.4)	0.006
	Yes	43 (9.1)	3(2.2)	46 (7.6)	
strangulation	No	457 (97.2)	139 (100)	596 (97.9)	0.047
	Yes	13 (2.8)	0 (0)	13(2.1)	
Nausea and vomiting	No	469 (99.8)	136 (97.8)	605 (99.3)	0.039
	Yes	1 (0.2)	3 (2.2)	4 (0.7)	
Urinary retention	No	444 (94.5)	138 (99.3)	582 (95.6)	0.015
	Yes	26 (5.5)	1 (0.7)	27 (4.4)	
Post operative complication	No	411(87.4)	119(85.6)	530 (87.0)	0.572
	Yes	59(12.6)	20(14.4)	79(13.0)	
Recurrence of hernia	No	465(98.9)	136(98.6)	601(98.8)	0.709
	Yes	5(1.1)	2(1.4)	7(1.15)	

Percentages were calculated using available data for each variable. Missing observations were excluded from individual analyses

**Table 4:** Type of surgery and continuous variables were compared using the Mann–Whitney U test

Variable	Open median (IQ)	Laparoscopic median (IQ)	*P*-value
AGE	55(41–66)	51.5(38–59)	< 0.001
Duration of surgery_min	113(88–140)	101.5(78-124.5)	< 0.001

Continuous variables are presented as median (IQR)

*P*-values were calculated using the Mann–Whitney U test

**Table 5:** Relation of anesthesia type with open repair and urinary retention

Anesthesia type (open repair)	Urinary retention	No urinary retention	Total
SA	12 (10.5%)	102 (89.5%)	114
GA	8 (3.8%)	202 (96.2%)	210
*p* = 0.027			

### Multivariable logistic regression

Multivariable logistic regression analysis was performed to identify factors associated with postoperative complications after inguinal hernia repair with mesh. The type of surgical approach (open versus laparoscopic) was not significantly associated with postoperative complications after adjustment for potential confounders (adjusted OR 1.22, 95% CI 0.07–20.18; *p* = 0.890). Similarly, age, sex, smoking status, marital status, heavy lifting, hypertension, diabetes mellitus, ischemic heart disease, past surgical history, previous groin surgery, symptom duration, anticoagulant or antiplatelet use, hernia type, laterality, unilateral versus bilateral disease, reducibility, hospital, operative technique subtype, mesh type, prophylactic antibiotic use, and postoperative pain score were not significantly associated with postoperative complications (all *p* > 0.05).

The presence of comorbidities was the only variable significantly associated with postoperative complications (adjusted OR 0.52, 95% CI 0.29–0.93; *p* = 0.028). Preoperative pain score demonstrated a borderline association with postoperative complications (adjusted OR 1.17, 95% CI 0.99–1.40; *p* = 0.070). Variables related to incarceration, obstruction, and strangulation produced unstable estimates due to sparse data and therefore, were not considered reliable predictors in the model (Table 6).

**Table 6:** Multivariable logistic regression analysis of factors associated with postoperative complications after mesh repair of inguinal hernia

Variable	Adjusted OR	95% CI	*P*-value
Type of procedure (open vs. laparoscopic)	1.22	0.07–20.18	0.890
Age (years)	0.998	0.975–1.022	0.876
Male sex	0.62	0.13–2.88	0.537
Smoking status (overall)	—	—	0.287
Current vs. never smoker	1.89	0.59–6.02	0.284
Ex-smoker vs. never smoker	2.94	0.77–11.25	0.115
Married	0.59	0.14–2.51	0.471
Heavy lifting history	1.27	0.52–3.10	0.601
Presence of comorbidities	0.52	0.29–0.93	0.028
Hypertension	0.99	0.48–2.07	0.980
Diabetes mellitus	1.35	0.57–3.17	0.495
Ischemic heart disease	0.64	0.21–1.90	0.418
Previous surgery (any type)	0.73	0.37–1.47	0.378
Previous groin surgery	1.07	0.51–2.25	0.857
Duration of symptoms (months)	1.00	0.995–1.007	0.811
Preoperative pain score (/10)	1.17	0.99–1.40	0.070
Anticoagulant/antiplatelet use	1.21	0.42–3.51	0.722
Type of inguinal hernia	0.95	0.61–1.49	0.829
Right vs. left hernia	0.83	0.48–1.44	0.508
Unilateral vs. bilateral	0.99	0.43–2.26	0.974
Reducible vs. irreducible	0.44	0.10–1.91	0.273
Hospital Type (private vs. governmental)	1.70	0.53–5.44	0.372
TAPP/TEP approach	0.88	0.21–3.60	0.856
Mesh type	0.89	0.52–1.50	0.652
Prophylactic antibiotic use	0.44	0.11–1.80	0.256
Postoperative pain score (/10)	1.61	0.83–3.14	0.161

## Discussion

As observed in the result section, a statistically significant association was found between type of surgery and type of inguinal hernia, with Direct hernias were more frequently treated using the laparoscopic approach, whereas indirect hernia were more commonly managed with open approach, this is consistent with previous studies which supports the use of laparoscopic technique in direct hernia more frequently because it is arise from weakness of posterior wall of inguinal canal, making them more suitable for placement of posterior mesh by laparoscopic technique, as laparoscopic allows better visualization of myopectinal orifice enabling precise reinforcement of the posterior wall. Also, direct hernia more commonly occurs bilaterally, and laparoscopic had a wide visualization to reveal occult contralateral defects [1, 5].

In contrast, indirect inguinal hernia in our cohort study was more commonly treated with an open approach; this trend is consistent with other studies, which show that indirect hernia occurs through the deep inguinal ring and may extend into the inguinal canal or scrotum. So large inguinoscrotal hernia, longstanding sac, and cases with severe adhesion can increase surgical technique difficulties and make the open approach more frequently used [1].

Patients presenting with complicated hernias ( incarceration, obstruction, and strangulation) were significantly more likely to undergo open repair. This is consistent with previous international studies and guidelines, which recommend open surgery in complicated cases due to the need for rapid access, assessment of bowel viability, and possible resection. However, laparoscopic repair may be feasible in selected cases; it is less commonly performed in emergency situations due to technical difficulties and concerns about pneumoperitoneum in compromised bowel [1]. These findings also highlight an important source of selection bias in the present study. Patients undergoing open repair had a higher proportion of complicated, irreducible, and strangulated hernias, whereas laparoscopic repair was predominantly performed in elective, uncomplicated cases. Therefore, differences observed between groups may partly reflect baseline differences in case complexity and surgeon selection rather than the effect of surgical approach alone. The results should therefore be interpreted with appropriate caution.

Similarly, Irreducible inguinal hernia were significantly more frequently treated with an open approach, which is consistent with previous studies, which report that irreducible hernia are more treated with open than laparoscopic herniorrhaphy [13]. This may be due to limited laparoscopic expertise and technical challenges in managing an irreducible inguinal hernia via laparoscopy.

As for a strangulated hernia, it is compatible with the irreducible hernia in management. However, in our study, we found that all patients with strangulation underwent open repair. This may be because time is critical, the operation must be performed as quickly as possible, allowing direct visualization and immediate management of bowel ischemia if present. This is more feasible with an open approach, especially since we do not have adequate facilities to prepare a laparoscopic theatre rapidly, nor sufficient surgical expertise for urgent laparoscopy [1, 13].

The overall postoperative complication (nausea and vomiting, urinary retention, surgical site infection, hematoma, seroma, mesh infection, scrotal edema, testicular complication, recurrence of hernia) was low and not significantly different between open and laparoscopic repair (12.6% vs. 14.4%, *p* = 0.572), which demonstrates the acceptable safety profile of both techniques. These findings are consistent with recent studies, which document comparable complication rates between open and laparoscopic hernia repair with mesh [14].

Despite a similar overall complication rate, certain postoperative events differed between the two approaches. Urinary retention was significantly more common with open procedures( *p* = 0.015), which may be related to the use of spinal anesthesia, age, and postoperative pain. Additional review of anesthesia records demonstrated that among patients undergoing open inguinal hernia repair, 114 received spinal anesthesia and 210 received general anesthesia. Postoperative urinary retention occurred in 12 of 114 patients (10.5%) who received spinal anesthesia compared with 8 of 210 patients (3.8%) who received general anesthesia. This difference was statistically significant (Fisher’s exact test, *p* = 0.027). All laparoscopic repairs (TAPP and TEP) were performed under general anesthesia. Previous studies have similarly identified spinal anesthesia and older age as important risk factors for postoperative urinary retention following inguinal hernia repair. On the other hand, nausea and vomiting were more frequent with laparoscopic procedures (*p* = 0.039), which may be attributed to pneumoperitoneum, peritoneal stretching, and the routine use of general anesthesia, all of which are established contributors to postoperative nausea and vomiting. Among laparoscopic repairs, 80.6% were performed using the TAPP approach. Because TAPP involves entry into the peritoneal cavity, the predominance of this technique may have contributed to the higher incidence of postoperative nausea and vomiting observed in the laparoscopic group. However, owing to the small number of events, a reliable subgroup analysis comparing TAPP and TEP procedures was not feasible [1, 14].

The findings of the present study should be interpreted as reflecting immediate and short-term postoperative outcomes rather than long-term surgical outcomes. While early postoperative complications can be assessed during the available follow-up period, the median follow-up duration of 7 days is insufficient to reliably evaluate recurrence, chronic postoperative pain, mesh-related complications, or long-term functional outcomes. In the current study, the overall early documented recurrence during available follow-up was uncommon; the recurrence rate was 1.2%, with 1.1% in the open group and 1.4% in the laparoscopic group [15, 16].

However, the median follow-up period of 7 days is not sufficient to assess the true recurrence rate, as recurrence usually takes months to years after surgery. Long-term follow-up studies have demonstrated that the recurrence rate increases with longer follow-up periods, with rates of 2–4% for both open and laparoscopic repairs [17, 18].

Thus, although early documented recurrence during the available follow-up period was uncommon, the short follow-up duration prevents reliable assessment of the true recurrence rate. Future prospective studies with longer follow-up are required to evaluate recurrence, chronic postoperative pain, and long-term mesh-related outcomes.

Also in this study, patient age was significantly associated with the choice of surgical technique. This is in line with new findings that show laparoscopic surgery is more often performed on younger which the median age of 51.5 (IQR 38–59), or more physiologically fit individuals, while open surgery is more often performed on older patients with a median age of 55 (IQR 41–66). Patients selected for laparoscopic surgery were, on average, younger than those undergoing open surgery, based on a recent nationwide study of elective inguinal hernia repairs [11]. To account for this potential confounding effect, age was included in the multivariable logistic regression model. After adjustment, age was not independently associated with postoperative complications, suggesting that the observed age difference alone did not explain postoperative outcomes.

The benefits of minimally invasive repair in older age groups have also been supported by recent studies that focus exclusively on geriatric patients. Laparoscopic inguinal hernia repair was associated with faster recovery and reduced postoperative and chronic pain without increasing the risk of complications, even in older patients, in a randomised controlled trial of patients aged ≥ 65 [19].

Unexpectedly and contrary to most previous studies, the open hernia mesh repair group required longer median operative time when compared to the laparoscopic hernia repair group (*p* = 0.001), meaning that open surgery took longer operative time (min).

One possible reason for this difference could be that the open approach in our setting could be more complicated as compared to the laparoscopic approach because it involves more tissue manipulation, it has a larger incision, and is used more in complicated and irreducible hernias. Secondly, the laparoscopic approach in our institution could be improved by optimized minimally invasive techniques and skilled personnel that could make it even shorter despite its technical challenge.

Comparatively, some more recent studies have shown that the operating time required for laparoscopic repair may be longer than that required by open repair. For example, a randomized controlled trial found that the mean operative time for laparoscopic inguinal hernia repair was significantly longer than for open mesh repair [20].

On the other hand, results from a large cohort of 107,073 patients showed that, while laparoscopic repair took longer than open repair under local anesthesia, the difference was slight under general anesthesia [12]. Although most randomized controlled trials report longer operative durations for laparoscopic repair, operative time is highly dependent on surgeon experience, case selection, and anesthesia type.

In this multicenter retrospective cohort study evaluating outcomes after mesh repair of inguinal hernia, we found that the type of surgical approach (open versus laparoscopic) was not independently associated with postoperative complications after adjustment for demographic, clinical, and operative variables. This finding suggests that baseline differences between groups, including age and hernia complexity, may have contributed to the unadjusted differences observed between surgical approaches. And also, this finding suggests that both techniques provide comparable short-term safety profiles when mesh repair is performed appropriately [21].

Several previous studies and systematic reviews have similarly reported no significant difference in overall postoperative complication rates between open and laparoscopic inguinal hernia repair, although laparoscopic approaches may offer advantages in postoperative recovery and chronic pain reduction in selected patients [22–24].

International hernia management guidelines also support a tailored approach to surgical technique selection, emphasizing that the choice between open and laparoscopic repair should be based on patient characteristics, hernia features, surgeon expertise, and available resources rather than on expectations of major differences in complication rates alone [21].

In the present study, comorbidity status emerged as the only factor significantly associated with postoperative complications in the multivariable model. Interestingly, the presence of comorbidities was associated with lower odds of postoperative complications. Although this finding reached statistical significance, it should be interpreted with caution because it contrasts with most surgical literature, where comorbid conditions are typically associated with increased postoperative risk [25].

One possible explanation for this observation is that patients with known comorbidities may have undergone more careful preoperative assessment, perioperative optimization, and postoperative monitoring, which may have mitigated complication risk. Alternatively, the finding may reflect residual confounding or selection bias inherent to retrospective observational studies [26].

Preoperative pain score demonstrated a borderline association with postoperative complications, suggesting that patients presenting with higher baseline symptom burden may represent a subgroup with more advanced disease or more complex operative conditions. Although this association did not reach statistical significance in the adjusted model, it may warrant further investigation in larger prospective cohorts [27].

Another important observation is the wide confidence interval observed for the procedure type variable, which indicates limited precision in estimating the true effect of surgical approach on postoperative complications. This may reflect relatively low event rates and sample size limitations, which are common challenges in observational surgical studies evaluating postoperative outcomes [26].

Overall, our findings suggest that patient-related factors may play a greater role than operative approach alone in determining postoperative outcomes after inguinal hernia repair. Consequently, individualized surgical decision-making that incorporates patient risk profile, hernia characteristics, and surgeon experience remains essential for optimizing outcomes.

### Limitations and strengths of the study

The current multicentre retrospective cohort study offers recent real-world surgical data for a comparison between open and laparoscopic inguinal hernia repair. One of the key strengths of the study is the fact that it has a fairly large number of participants (*n* = 609), which helps to make the results more robust. The fact that it is a multicentre study helps to increase its generalizability. Another strength is that mesh repair was used in all cases, which helps to standardize the procedure according to the recent international guidelines for groin hernia repair, which recommend mesh repair as the standard of care. The study takes into account a wide range of perioperative parameters, which helps to assess the data in terms of operative time, early postoperative complications, and length of stay, which are considered to be short-term quality indicators in hernia surgery.

However, there are a few limitations that need to be considered. The retrospective and non-randomized nature of the study introduces an inherent risk of selection bias. Additionally, patients undergoing laparoscopic repair were significantly younger than those undergoing open repair, which may reflect age-related selection bias in surgical decision-making. However, age was included in the multivariable regression model and was not independently associated with postoperative complications.

Patients undergoing open repair had a substantially higher proportion of complicated, irreducible, and strangulated hernias, whereas laparoscopic repair was more commonly performed in elective, uncomplicated cases. Consequently, the two groups were not fully comparable at baseline, and differences in outcomes may partly reflect case selection and surgeon preference rather than the surgical approach itself. The imbalance in the number of patients in both groups (open surgery 77% vs. laparoscopic 23%) is likely due to institutional preference and resource availability. Additionally, the median follow-up period of 7 days is too short to allow reliable evaluation of long-term outcomes such as recurrence, chronic postoperative inguinal pain, mesh-related complications, and long-term functional recovery. Therefore, the findings of this study should primarily be interpreted as short-term postoperative outcomes rather than long-term measures of surgical success. Missing values in certain variables may also have impacted the accuracy of the analysis. In addition, operative details such as mesh fixation strategy and surgeon-specific technical variations were not consistently documented in the medical records. Therefore, complete standardization of laparoscopic techniques across surgeons and participating centers could not be verified. Also, we couldn’t accurately evaluate chronic pain in patients, and didn’t make a cost analysis between the two approaches. Although multivariable logistic regression was performed, residual confounding cannot be excluded. In multivariable logistic regression, variables related to incarceration, obstruction, and strangulation yielded unstable regression estimates due to sparse event counts (quasi-complete separation) and were therefore interpreted with caution. Prospective studies with longer follow-up periods are needed for the assessment of rates of recurrence, chronic pain, functional results, and cost analysis.

## Conclusion

This large retrospective cohort study of 609 patients provides a comprehensive comparison of the differences in outcomes between open and laparoscopic inguinal hernia repair with mesh. Open repair was the predominant technique, accounting for more than three-quarters of cases, while laparoscopic repair was performed in a selected subset of patients.

Significant associations were observed between surgical type approach and hernia characteristics. Laparoscopic repair was more frequently performed in direct and reducible hernias and in patients without complications, whereas open repair was more commonly used in indirect, irreducible, strangulated, and complicated hernias. Patients undergoing laparoscopic repair were significantly younger, and the laparoscopic approach was associated with a shorter operative duration.

Overall postoperative complication rates were low and comparable between the two techniques. Early documented recurrence during the available follow-up period was uncommon and did not differ significantly between groups; however, the short follow-up duration precludes reliable assessment of true recurrence rates and other long-term outcomes. Taken together, both open and laparoscopic mesh repair demonstrated favourable safety profiles and satisfactory short-term postoperative outcomes. Further prospective studies with longer follow-up are required to evaluate recurrence, chronic postoperative pain, mesh-related complications, and long-term patient-centred outcomes. These findings support the use of either approach depending on clinical presentation, surgeon expertise, institutional resources, and operative planning, with both techniques offering safe and effective repair.

Further studies are needed to compare the cost of the two surgeries, the need for drain insertion, and the surgeon’s experience, which is recommended to enhance surgical decision-making in the Palestinian hospital.

## Data Availability

The datasets used and/or analysed during the current study are available from the corresponding author on reasonable request.
